# Pulsed electric field performance calculator tool based on an *in vitro* human cardiac model

**DOI:** 10.3389/fphys.2024.1395923

**Published:** 2024-06-07

**Authors:** Maura Casciola, Abouzar Kaboudian, Tromondae K. Feaster, Akshay Narkar, Ksenia Blinova

**Affiliations:** Office of Science and Engineering Laboratories, Center for Devices and Radiological Health, United States Food and Drug Administration, Silver Spring, MD, United States

**Keywords:** pulsed field ablation, electroporation, lethal electric field threshold, human induced pluripotent stem cell-derived cardiomyocytes, preclinical testing, cardiac ablation technologies, online tool, non-thermal ablation

## Abstract

**Introduction:**

Pulsed Field Ablation (PFA) is a novel non-thermal method for cardiac ablation, relying on irreversible electroporation induced by high-energy pulsed electric fields (PEFs) to create localized lesions in the heart atria. A significant challenge in optimizing PFA treatments is determining the lethal electric field threshold (EFT), which governs ablation volume and varies with PEF waveform parameters. However, the proprietary nature of device developer’s waveform characteristics and the lack of standardized nonclinical testing methods have left optimal EFTs for cardiac ablation uncertain.

**Methods:**

To address this gap, we introduced a laboratory protocol employing human induced pluripotent stem cell-derived cardiomyocytes (hiPSC-CMs) in monolayer format to evaluate the impact of a range of clinically relevant biphasic pulse parameters on lethal EFT and adiabatic heating (AH). Cell death areas were assessed using fluorescent dyes and confocal microscopy, while lethal EFTs were quantified through comparison with electric field numerical simulations.

**Results and conclusion:**

Our study confirmed a strong correlation between cell death in hiPSC-CMs and the number and duration of pulses in each train, with pulse repetition frequency exerting a comparatively weaker influence. Fitting of these results through machine learning algorithms were used to develop an open-source online calculator. By estimating lethal EFT and associated temperature increases for diverse pulse parameter combinations, this tool, once validated, has the potential to significantly reduce reliance on animal models during early-stage device de-risking and performance assessment. This tool also offers a promising avenue for advancing PFA technology for cardiac ablation medical devices to enhance patient outcomes.

## 1 Introduction

Pulsed Field Ablation (PFA) or Pulsed Electric Field (PEF) catheter ablation devices have emerged as promising “non-thermal” alternatives for treating patients with antiarrhythmic drug-resistant atrial fibrillation (AF), currently advancing through clinical development (Reddy et al., 2021; Ekanem et al., 2022; Verma et al., 2022; Verma et al., 2023). PFA represents a newer and potentially less invasive approach compared to traditional thermal catheter ablation techniques (Reddy et al., 2018; Maan and Koruth, 2022; Reddy et al., 2023), aiming to enhance safety and reduce off-target tissue effects while creating predictable long-lasting lesions (Reddy et al., 2020; Nakatani et al., 2021; Verma et al., 2021).

PFA involves delivering high-energy electrical pulses to induce localized lesions in the atria of the heart. These lesions, achieved through irreversible cell electroporation, disrupt abnormal electrical pathways responsible for irregular heart rhythms. Electroporation, the augmentation of cell permeability, can be localized by intensifying the electric field in the target region (Maor et al., 2019; Sugrue et al., 2019). The lethal electric field threshold (EFT) for a given tissue signifies the minimum electric field strength required for inducing irreversible cell permeabilization (Davalos et al., 2005). When the electric field intensity surpasses this threshold, changes in cell homeostasis produced by cell permeabilization lead to cell death (Sweeney et al., 2016; Peng et al., 2023) without harm to the extracellular matrix. Notably, the selection of PEF waveform parameters at the lethal EFT is a critical factor in understanding and controlling the irreversible electroporation phenomenon. In fact, this threshold varies depending not only on the cell type and size, but also on the specific waveform parameters selected (e.g., pulse amplitude, duration, number, repetition frequency, shape, and train number) (Miklavcic and Davalos, 2015; Sano et al., 2018; Aycock and Davalos, 2019) impacting the treatment outcomes and lesion predictability (Flisar et al., 2003; Weaver et al., 2012; Miklovic et al., 2017).

Characterizing the lethal EFT for specific tissues and PEF treatments is fundamental for the safe and successful application of PFA-based medical procedures. Quantifying lethal EFT as a function of waveform parameters is essential to optimize electroporation techniques, striking a balance between achieving the desired membrane damage and minimizing potential side effects, including undesired Joule heating, neuromuscular stimulation, bubble formation, coronary spasm, or damage (Rubinsky et al., 2008; Nickfarjam and Firoozabadi, 2014; Valerio et al., 2017; Sugrue et al., 2019; Aycock et al., 2022; Gudvangen et al., 2022). Yet, despite the potential of PFA, the establishment of optimal lethal EFTs for cardiac ablation remains inconclusive. Moreover, limited data are available regarding the impact of waveform parameter selection on cardiac lesion formation, largely due to medical device developers with proprietary waveform characteristics leading the PFA development. Only a handful of nonclinical studies have published the waveform parameters used (Stewart et al., 2019; van Es et al., 2019; Caluori et al., 2020; Loh et al., 2020), or investigated the impact of these parameters on treatment effectiveness in diverse animal models (Zager et al., 2016; Heller et al., 2021; Yavin et al., 2021; García-Sánchez et al., 2022; Kos et al., 2023).

The absence of standardized nonclinical testing methods, grounded in human physiology, for predicting the lethal EFT and resultant ablation lesion induced by a specific PEF waveform selection, coupled with varying catheter geometries, has led to suboptimal design and a rise in animal usage in device development. Presently, PFA device developers rely on in-house nonclinical bench tests for initial safety and effectiveness assessments. Nevertheless, the diversity in experimental conditions and testing methods complicates the regulatory evaluation of these devices. The establishment of standardized nonclinical evaluation tools for PFA systems could accelerate treatment optimization and facilitate regulatory assessments, ultimately ensuring life-saving therapies reach patients in need.

In recent years, *in vitro* platforms utilizing human induced pluripotent stem cell-derived cardiomyocytes (hiPSC-CMs) have emerged as versatile tools for nonclinical drug screening, development (Blinova et al., 2019) and the evaluation of cardiac electrophysiology medical devices (Feaster et al., 2021; Feaster et al., 2022). Due to their similarity to adult human cardiac tissue, hiPSC-CMs offer a solution that minimizes species and biological disparities present in other cardiac models, while reducing the reliance on large animals (Yang et al., 2014; Ribeiro et al., 2015). In a prior study, a standardized laboratory protocol was introduced for evaluating and optimizing PEF cardiac ablation parameters using hiPSC-CMs in a high-throughput monolayer format (Casciola et al., 2023). This study systematically explored a wide range of pulse parameters demonstrating that hiPSC-CMs respond to changes in pulse amplitude, duration, number, and repetition frequencies by irreversible electroporation. We systematically varied one parameter at a time quantifying lethal EFTs, reversible electroporation effects, potential thermal impacts under adiabatic conditions, total treatment time, and absorbed doses for each waveform combination. The selection of optimal pulse parameters within the studied range aimed to minimize lethal EFT, thereby avoiding extraneous stimulation, tissue overheating, and prolonged procedures while delivering effective treatment. Once validated, this hiPSC-CMs-based assay for PEF cardiac ablation assessment, could inform treatment parameter selection for PFA devices by providing parameter dependent lethal EFTs.

In the present study, we extend these findings to cover higher pulse repetition frequency (PRF) commonly used in preclinical and clinical studies (Zager et al., 2016; Stewart et al., 2019; van Es et al., 2019; Caluori et al., 2020; Loh et al., 2020; Heller et al., 2021; Yavin et al., 2021; García-Sánchez et al., 2022), and shorter pulse durations to meet emerging nanosecond PEF approaches (Varghese et al., 2023/02). Experimental results were fitted in a multivariable domain to obtain interpolated data that have been used to create an online, open-source tool (GitHub - dbp-osel/PFACalculatorTool: This tool is developed to estimate the lethal PFA from input pulse parameters), enabling the calculation of lethal EFT and adiabatic temperature increases for any combination of pulse parameters within the studied range. Such a tool offers potential benefits in early device safety and effectiveness assessment, reducing reliance on animal models and expediting progress in the field.

## 2 Materials and methods

### 2.1 Cell culture and maintenance

A commercially available mixed population of HiPSC-CMs, iCell Cardiomyocytes^2^ catalog # 01434 (Fujifilm Cellular Dynamics, Inc., Madison, WI), were handled as described in detail in Casciola et al. (2023). Briefly, hiPSC-CMs, in a concentration of 120,000 cells per well, were plated on 96-well Nanofiber plates, catalog # 9602 (Nanofiber Solutions, Dublin, OH), coated with Matrigel substrate, catalog # 356230 (Corning Inc., Somerville, MA), in a 1:60 DMEM dilution, catalog # 30-2006 (ATCC, Manassas, VA). Cells were maintained using iCell Cardiomyocytes Maintenance Medium, catalog # M1003 (Fujifilm Cellular Dynamics, Inc.). Spontaneously beating, 100% confluent hiPSC-CM monolayers were used for PEF treatment testing on days 4–7 after plating. iCell Cardiomyocytes Serum-Free Medium (iCM-SF), catalog # M1038 (Fujifilm Cellular Dynamics, Inc.), was used during cell incubation between PEF application and imaging. Modified Tyrode’s containing Calcein-AM and Propidium Iodide was used during pulsing and imaging.

### 2.2 PEF treatments delivery

A pair of custom stainless-steel needle electrodes (0.7 mm diameter, 1.72 mm distance center-to-center) were connected to an electric pulse generator, model pulse generator EPULSUS-FBM1-5 (EnergyPulse Systems, Lda., Portugal). A 3D printer, model Anet A8 (Shenzhen Anet Technology Co., China), was used as an automated robotic arm for accurate positioning of the electrodes perpendicular to the cell monolayer. The 3D printer was programmed to move the electrodes onto the center of each well bottom with a 10 s delay. Its stage was heated to 50°C to adjust the pretreatment temperature of the Tyrode solution to 37.5°C ± 1.0°C. A multi-well plate holder (Olympus, Center Valley, PA) was fixed to the 3D printer stage to avoid relative movements of the multi-well plate. Pulse shape and amplitude were measured with an isolated oscilloscope (Tektronix, Beaverton, OR) connected to a 1:100 voltage probe, model P2501 (Owon Technology Inc., China).

**FIGURE 1:**
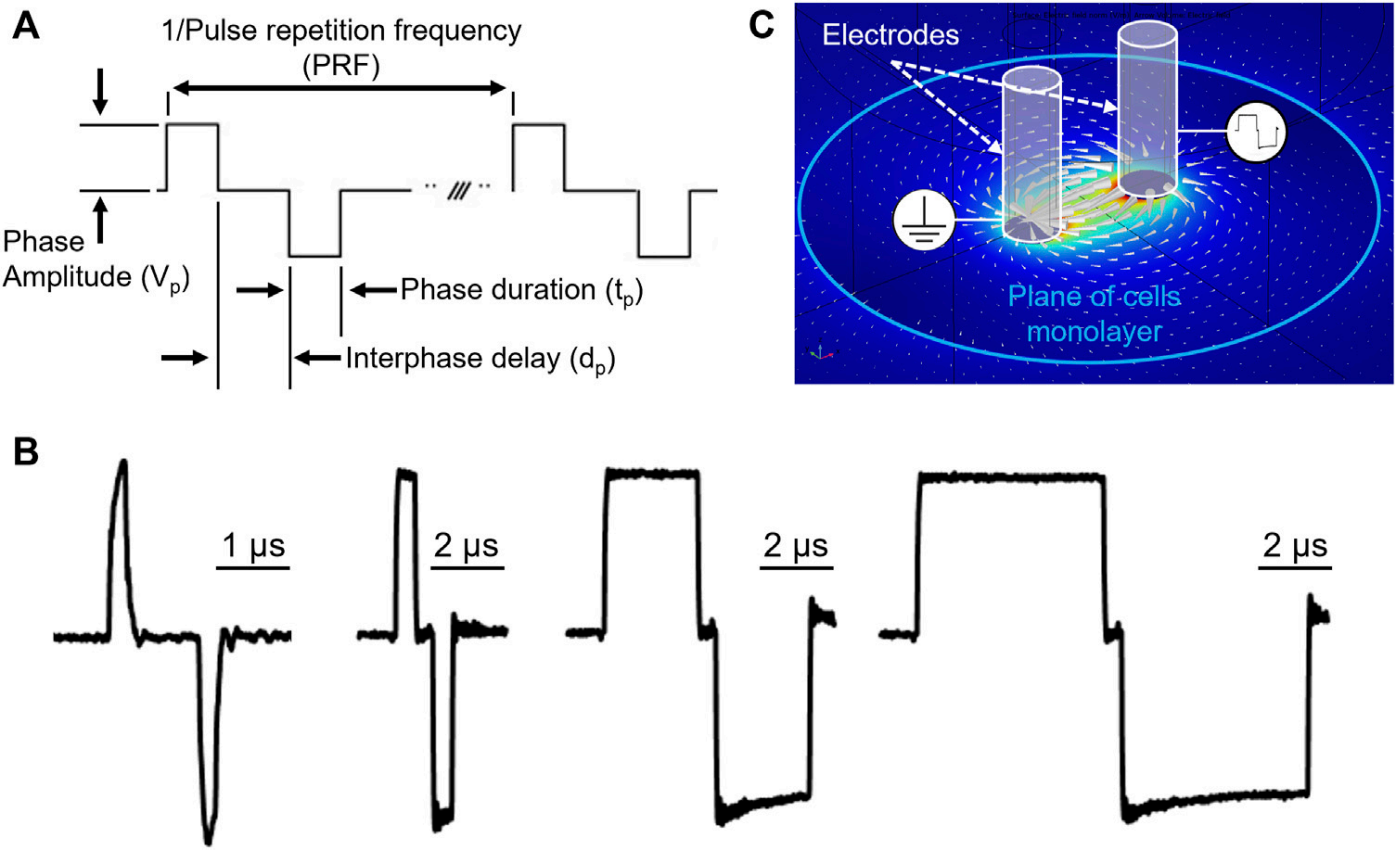
PEF waveform description and electric field map in the cell monolayer plane. **(A)** Ideal voltage waveform, and relevant PEF parameters definitions. The pulse repetition frequency is the inverse of the biphasic pulse repetition period. **(B)** Representative biphasic pulses used in study with phase duration 0.2, 1, 5, 10 µs respectively, measured at the stimulating electrodes immerged in the working solution. **(C)** Electric field distribution generated by the stimulating electrodes in an environment mimicking experimental conditions.

The biphasic electrical pulses were described by the following parameters (Figure 1A): pulse repetition frequency (PRF); number of biphasic pulses (p_#_) delivered in a single train; phase amplitude (V_p_); phase duration (t_p_); and interphase delay (d_p_). For each phase duration a representative bipolar pulse is shown in Figure 1B. For each combination of PEF parameters, V_p_ was gradually increased until a clear measurable electroporation region was produced, while avoiding cell detachment and monolayer dissociation (Casciola et al., 2023). The interphase delay was maintained for all the PEF combinations at 1 µs.

### 2.3 Fluorescence imaging

Hoechst-33342 (Ho) [2.25 µM], catalog # H3570 (Invitrogen, ThermoFisher Scientific, Waltham, MA) dye, labeling the nuclei of all cells, was used to assess monolayer confluency and integrity before and after pulsing. PEF-treated hiPSC-CM monolayers were stained with two cell-impermeable fluorescent probes: Calcein-AM [2 µM], live cell indicator, and Propidium Iodide (PI) [15 µM], irreversible electroporated cell indicator, catalog numbers C3100MP and P3566, respectively (Invitrogen, ThermoFisher Scientific), at different times (Supplementary Figure S1). Fifteen minutes before experiment, cells were washed with 200 µL per well of DPBS, catalog number 14190-144 (Gibco, ThermoFisher Scientific). DPBS was immediately replaced with 100 µL per well of modified Tyrode solution containing Calcein-AM. 30 min after PEF treatments, the Tyrode solution was changed to iCM-SF, 100 µL per well, and plates were returned to a 37°C, 5% CO_2_ cell culture incubator. To stain dead cells, iCM-SF was substituted with Tyrode containing PI 15 min prior imaging 2–4 h after PEF treatment, as optimized in Casciola et al. (2022) and Casciola et al. (2023).

The laser scanning confocal microscope, model FluoView 3000 (Olympus) equipped with an environmental chamber (i.e., 37°C and 5% CO_2_), model OKO-H301-OLY-IX3-SVR (Okolab stl, Italy), was used to collect fluorescent images from multiple wells using a 4X, NA/0.16 dry objective. Four images per well were acquired and stitched prior to analysis.

### 2.4 Analysis of cell death areas

To quantify the cell death area, Calcein-AM and PI-stained regions were analyzed with ImageJ software (NIH, Bethesda, MD) (Schneider et al., 2012) similarly to Casciola et al. (2022), with the only difference that Calcein-AM stained images contrast was enhanced one time through ImageJ automated feature. The imprint area of each electrode was quantified from sham exposures and subtracted from the electroporated area when needed. When the Analyze Particles tool failed to provide area measurements, the wand feature of ImageJ or manual selection was used to identify the edges of the cell death region and quantify its surface (Liu et al., 2021).

### 2.5 Numerical simulations and estimation of electroporation and lethal EFTs

The external edge of the cell death region provides the EFT for lethal effects. As described in Casciola et al. (2023), lethal EFTs were identified by comparison of the areas stained with Calcein-AM and PI to the electric field map from numerical simulations (Arena et al., 2012; Neal et al., 2014; Aycock et al., 2021; Liu et al., 2021; Aycock et al., 2022). The finite element analysis software Comsol Multiphysics 5.6 (COMSOL Inc., Stockholm, Sweden) was used to compute in static conditions the applied electric field distribution in the cell monolayer plane (Figure 1C). The experimental setup was modeled as reported in Casciola et al. (2023) except for the electrode diameter and center-to-center distance, modified to match the current geometry adopted for this study. For other details on materials, geometry and lethal EFT estimation from fluorescence images see Casciola et al. (2023).

To estimate the temperature increase in adiabatic conditions, the adiabatic heating (AH, °C) was derived from the absorbed dose (AD, mJ/g) function of the EFT obtained by numerical modeling and calculated as reported in Casciola et al. (2023).

### 2.6 Data fitting and PFA *in vitro* performance calculator

A log-log regression model was trained to determine the dependence of EFT on the number of pulses, phase duration, and pulse repetition frequency. To construct this log-log regression, scikit-learn python library was utilized (Pedregosa et al., 2011). From the observed features, namely, number of pulses, phase duration, and pulse repetition frequency, logarithmic features were constructed. Similarly, the logarithmic transform of the observed lethal EFTs was calculated. All the logarithmic features as well as the log of observed lethal EFTs were passed to the Linear Regression model in scikit-learn for fitting. The fitting in this model was carried out by minimizing the residual sum of squares between the experimentally observed and model predicted lethal EFT (Hackeling, 2017). A 10-fold cross validation was performed in each case to ensure the quality of the fit (Fushiki, 2011) and the coefficient of determination (*R* ^2^) for the training and test sets were calculated. The resulting log-log fit was observed to provide a *R* ^2^ without overfitting the data for the training sets ( $R^{2}=0.954\pm 0.002$ ) and the test sets ( $R^{2}=0.949\pm 0.017$ ). This log-log fit was subsequently used to create a calculator tool that can estimate the EFT and the AH based on the user’s input number of pulses, phase duration, and pulse repetition frequency.

### 2.7 Statistical analysis

Results are presented as mean ± standard error. Cell death areas, as well as derived endpoints for a given set of PEF parameters were calculated as an average for *n* = 4–8 per group.

## 3 Results

### 3.1 Calcein-AM and propidium iodide as markers of area in HiPSC-CM monolayers

**FIGURE 2:**
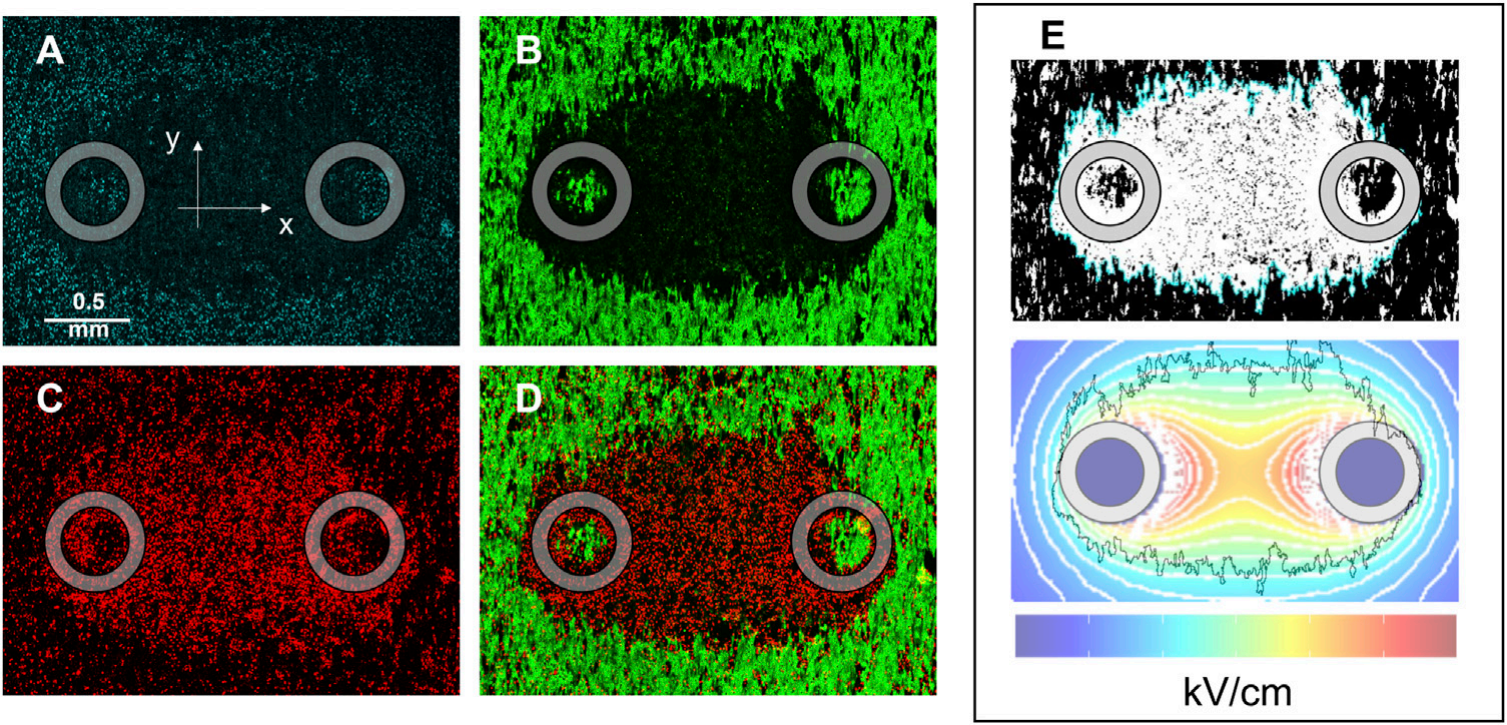
HiPSC-CM monolayer staining and lethal EFT quantification. Images were acquired 2–4 h after PEF treatment to evaluate cell death by electroporation. Fluorescent dyes were used to stain: **(A)** Cell nuclei, Hoechst-33342; **(B)** live cells, Calcein-AM; **(C)** dead cells, Propidium Iodide; **(D)** overlay of green and red channels showed PI-stained cells (PEF induced membrane permeabilization) surrounding the electrodes and an external Calcein-AM-stained region (cell surviving the PEF treatment). **(E)** Analysis was performed for Calcein-AM and PI staining at the end of the experiments to compare the areas identified by fluorescence staining to the area identified by electric field isolines (light blue). The black jagged border outline in the lower panel of **(E)** corresponds to the light blue border outline in the upper panel of **(E)** and represents the border of the cell death area. Gray circles indicate the footprints of the electrodes positioned orthogonally to the cell monolayer during PEF treatment.

The effects of PEF treatments were assessed in hiPSC-CMs by measuring the area of Calcein-AM and Propidium Iodide (PI) uptake in the monolayer. Cell monolayers were imaged 2–4 h after treatment, and staining with Hoechst, Calcein-AM, and PI was recorded (Figures 2A–C, respectively). PI becomes fluorescent when it enters cells following PEF-induced permeabilization, while Calcein-AM selectively stains live cells that have survived the treatment, leaving a darker area around the pulse-delivering electrodes, which corresponds to the region of cell death (Figure 2D). The areas obtained from the analysis of both types of images were quantified and converted into lethal EFT values (Figure 2E).

Within the range of waveform parameters investigated, both cell death areas and lethal EFT values obtained from Calcein-AM and PI staining yielded comparable results (Table 1). Specifically, a detailed analysis of the experiments on the 100 pulses per trains revealed that while the values of cell death areas (Table 2) and corresponding EFTs (Supplementary Figure S2) were statistically equivalent, automated image analysis demonstrated a higher success rate with Calcein-AM staining as compared to PI. Therefore, for all subsequent experiments described in this study, EFT values were calculated based on Calcein-AM stained images.

**TABLE 1:** PEF treatment endpoints for the range of PEF parameters investigated in hiPSC-CMs.

p_#_	t_p_ (µs)	V_p_ (V)	PRF (Hz)	PI area (mm^2^)	PI EFT (kV/cm)	CA area (mm^2^)	CA EFT (kV/cm)	AH (°C)
50	0.2	1,490	200	1.18 ± 0.18	7.65 ± 0.29	1.18 ± 0.28	7.66 ± 0.44	6.48 ± 0.75
			50	1.26 ± 0.3	7.54 ± 0.47	1.2 ± 0.11	7.63 ± 0.18	6.42 ± 0.29
			20	1.3 ± 0.47	7.47 ± 0.71	1.33 ± 0.36	7.42 ± 0.57	6.1 ± 0.95
			2	1.55 ± 0.34	7.09 ± 0.5	1.5 ± 0.35	7.17 ± 0.52	5.69 ± 0.81
	1	660	200	1.8 ± 0.75	3.41 ± 0.74	1.81 ± 0.82	3.53 ± 0.87	6.43 ± 3.11
			50	2.41 ± 1.4	2.93 ± 1.09	2.29 ± 0.95	3.09 ± 0.83	5.49 ± 3.55
			20	2.29 ± 0.51	2.97 ± 0.36	2.18 ± 0.41	3.15 ± 0.35	5.33 ± 1.38
			2	1.79 ± 0.7	3.52 ± 0.7	1.71 ± 0.66	3.6 ± 0.69	7.23 ± 2.77
	5	280	50	1.21 ± 0.34	1.85 ± 0.17	1.18 ± 0.53	1.87 ± 0.26	9.66 ± 2.56
			20	1.28 ± 0.23	1.81 ± 0.12	1.25 ± 0.3	1.82 ± 0.15	9.21 ± 1.46
			2	2.13 ± 0.32	1.44 ± 0.13	2.16 ± 0.84	1.43 ± 0.35	5.73 ± 2.84
	10	198	20	1.71 ± 1.07	1.11 ± 0.37	1.66 ± 0.88	1.12 ± 0.34	7.13 ± 4.19
			2	2.04 ± 1.1	0.99 ± 0.34	2.15 ± 0.82	0.95 ± 0.25	5.06 ± 2.7
100	0.2	1,380	200	2.24 ± 0.59	5.83 ± 0.72	2.57 ± 0.43	5.46 ± 0.46	6.6 ± 1.13
			50	1.9 ± 0.55	6.25 ± 0.74	1.95 ± 0.23	6.17 ± 0.29	8.41 ± 0.77
			20	1.88 ± 0.66	6.28 ± 0.85	1.78 ± 0.24	6.4 ± 0.32	9.06 ± 0.89
			2	1.95 ± 0.58	6.2 ± 0.76	2.01 ± 0.53	6.12 ± 0.63	8.3 ± 1.64
	1	590	200	3.25 ± 1.03	2.14 ± 0.52	2.99 ± 0.4	2.27 ± 0.23	5.68 ± 1.13
			50	3.59 ± 1.76	2.02 ± 0.78	3.43 ± 0.59	2.04 ± 0.28	4.6 ± 1.23
			20	2.53 ± 0.85	2.26 ± 0.49	2.49 ± 0.61	2.28 ± 0.36	5.76 ± 1.71
			2	2.17 ± 0.61	2.5 ± 0.47	2.19 ± 0.77	2.49 ± 0.6	6.96 ± 3.34
	5	256	50	2.2 ± 0.89	1.23 ± 0.32	2.25 ± 1.1	1.22 ± 0.4	8.39 ± 5.29
			20	2.54 ± 1.65	1.13 ± 0.51	2.77 ± 1.52	1.07 ± 0.41	6.63 ± 4.68
			2	1.88 ± 0.61	1.08 ± 0.21	1.59 ± 0.59	1.18 ± 0.24	7.84 ± 3.15
	10	170	20	2.31 ± 0.77	0.76 ± 0.15	2.25 ± 0.57	0.77 ± 0.13	6.59 ± 2.21
			2	2.88 ± 0.47	0.65 ± 0.08	2.97 ± 0.61	0.64 ± 0.1	4.48 ± 1.3
200	0.2	880	200	1.5 ± 0.36	4.83 ± 0.44	1.35 ± 0.4	5.01 ± 0.51	11.12 ± 2.23
			50	1.97 ± 0.34	4.28 ± 0.36	1.69 ± 0.49	4.6 ± 0.57	9.39 ± 2.29
			20	1.81 ± 0.46	4.46 ± 0.52	1.69 ± 0.57	4.6 ± 0.66	9.42 ± 2.68
			2	2.07 ± 0.68	4.2 ± 0.7	1.92 ± 0.51	4.35 ± 0.54	8.38 ± 2.05
	1	356	200	1.46 ± 0.51	2.12 ± 0.37	1.31 ± 0.33	2.23 ± 0.28	11.02 ± 2.71
			50	2.19 ± 0.53	1.65 ± 0.27	2.23 ± 0.57	1.64 ± 0.28	5.98 ± 2.01
			20	2.09 ± 0.84	1.73 ± 0.41	1.99 ± 0.78	1.69 ± 0.5	7.03 ± 3.36
			2	2.4 ± 1.35	1.6 ± 0.52	2.31 ± 0.98	1.62 ± 0.41	5.91 ± 2.72
	5	190	50	1.63 ± 0.48	1.08 ± 0.19	1.55 ± 0.41	1.11 ± 0.17	13.61 ± 4.25
			20	1.87 ± 0.58	0.99 ± 0.19	1.72 ± 0.32	1.04 ± 0.12	11.97 ± 2.63
		140	2	1.77 ± 0.4	0.79 ± 0.09	1.63 ± 0.36	0.82 ± 0.08	7.51 ± 1.42
	10	132	20	1.89 ± 0.73	0.73 ± 0.15	1.99 ± 0.85	0.71 ± 0.18	11.49 ± 5.6
		100	2	2.24 ± 1.2	0.49 ± 0.21	2.43 ± 1.02	0.46 ± 0.16	4.74 ± 2.89
400	0.2	584	200	1.22 ± 0.15	3.75 ± 0.19	0.97 ± 0.14	4.07 ± 0.18	14.65 ± 1.3
			50	1.14 ± 0.22	3.85 ± 0.29	0.91 ± 0.28	4.16 ± 0.39	15.32 ± 2.83
			20	0.98 ± 0.32	4.07 ± 0.43	0.92 ± 0.42	4.15 ± 0.59	15.28 ± 4.37
			2	1.17 ± 0.44	3.81 ± 0.53	0.82 ± 0.38	4.29 ± 0.54	16.31 ± 4.1
	1	312	200	1.5 ± 0.36	1.26 ± 0.12	1.51 ± 0.64	1.26 ± 0.21	7.09 ± 2.24
	1	280	50	0.73 ± 0.47	2.13 ± 0.3	0.9 ± 0.42	2.01 ± 0.25	17.96 ± 4.3
			20	1.2 ± 0.11	1.85 ± 0.06	0.92 ± 0.21	2 ± 0.12	17.69 ± 2.05
			2	1.77 ± 0.79	1.59 ± 0.33	1.42 ± 0.4	1.74 ± 0.19	13.46 ± 2.77
	5	122	50	1 ± 0.32	0.88 ± 0.09	0.84 ± 0.35	0.92 ± 0.09	18.79 ± 3.5
		100	20	1.16 ± 0.52	0.71 ± 0.14	0.64 ± 0.47	0.85 ± 0.14	16.17 ± 4.9
			2	1.89 ± 0.6	0.55 ± 0.12	1.98 ± 0.52	0.53 ± 0.1	6.25 ± 2.03
	10	90	20	1.49 ± 0.51	0.57 ± 0.12	1.38 ± 0.68	0.59 ± 0.16	15.74 ± 7.88
		55	2	1.56 ± 0.52	0.33 ± 0.08	1.28 ± 0.54	0.38 ± 0.09	6.41 ± 2.96

For all combinations of waveform parameters tested, we reported: cell death areas and lethal EFTs for estimated by analysis of Calcein-AM (CA) and PI images, the calculated adiabatic heating (AH). For all the endpoints tabled, we reported average and standard error. *N* = 4–8.

**TABLE 2:** Area estimated by Calcein-AM and PI and success rates with automated and manual area selection.

p#	t_p_ (µs)	PRF (Hz)	CA area (mm^2^)	PI area (mm^2^)	*p*-value	CA automated analysis #areas/#Experiments	PI automated analysis #areas/#Experiments	PI manual analysis #areas/#Experiments
100	0.2	200	5.46 ± 0.12	5.83 ± 0.18	0.11	6/6	6/6	0/6
		50	6.15 ± 0.09	6.24 ± 0.19	0.65	6/6	5/6	1/6
		20	6.38 ± 0.09	6.27 ± 0.22	0.65	6/6	4/6	1/6
		2	6.12 ± 0.18	6.20 ± 0.19	0.78	8/10	7/10	3/10
	1	200	2.27 ± 0.07	2.13 ± 0.13	0.39	6/6	4/6	3/6
		50	2.08 ± 0.07	2.02 ± 0.20	0.78	6/6	5/6	3/6
		20	2.24 ± 0.10	2.25 ± 0.13	0.96	6/6	5/6	0/6
		2	2.49 ± 0.15	2.50 ± 0.12	0.98	5/6	5/6	5/6
	5	50	1.22 ± 0.10	1.27 ± 0.09	0.67	5/6	NA	6/6
		20	1.14 ± 0.13	1.13 ± 0.13	0.98	6/6	4/6	3/6
		2	1.18 ± 0.06	1.08 ± 0.06	0.2	6/6	6/6	4/6
	10	20	0.79 ± 0.04	0.76 ± 0.04	0.61	6/6	5/6	4/6
		2	0.63 ± 0.01	0.65 ± 0.02	0.4	4/6	NA	2/6

For 100# pulse PEF treatments tested, we reported: cell death area estimated by analysis of Calcein-AM (CA) and PI images, and success rate with automated and manual area selection. Manual selection included the use of the wand feature of ImageJ or user manual identification of the cell death edge. The average and standard error were calculated for Calcein-AM and PI images obtained from the same wells treated, i.e., if for the same pulsing condition, the analysis on Calcein-AM images resulted in more data than from PI images, only the Calcein-AM values corresponding to PI analysis were considered.

### 3.2 Effect of PEF waveform parameters on the lethal EFT in HiPSC-CMs

The dependence of lethal EFTs on PEF waveform characteristics was investigated by varying one parameter at a time in independent experiments conducted on hiPSC-CM monolayers and assessed by Calcein-AM staining. Single trains of biphasic pulses with varying parameters were generated, with 50, 100, 200, and 400 pulses per train, and phase durations of 0.2, 1, 5, and 10 µs. These pulses were delivered at various PRFs including 2, 20, 50, and 200 kHz. The results demonstrated a clear dependence of the cell death area (Table 1; Supplementary Figure S3) and the lethal EFT (Table 1; Supplementary Figure S4) with changes in the PEF waveform.

**FIGURE 3:**
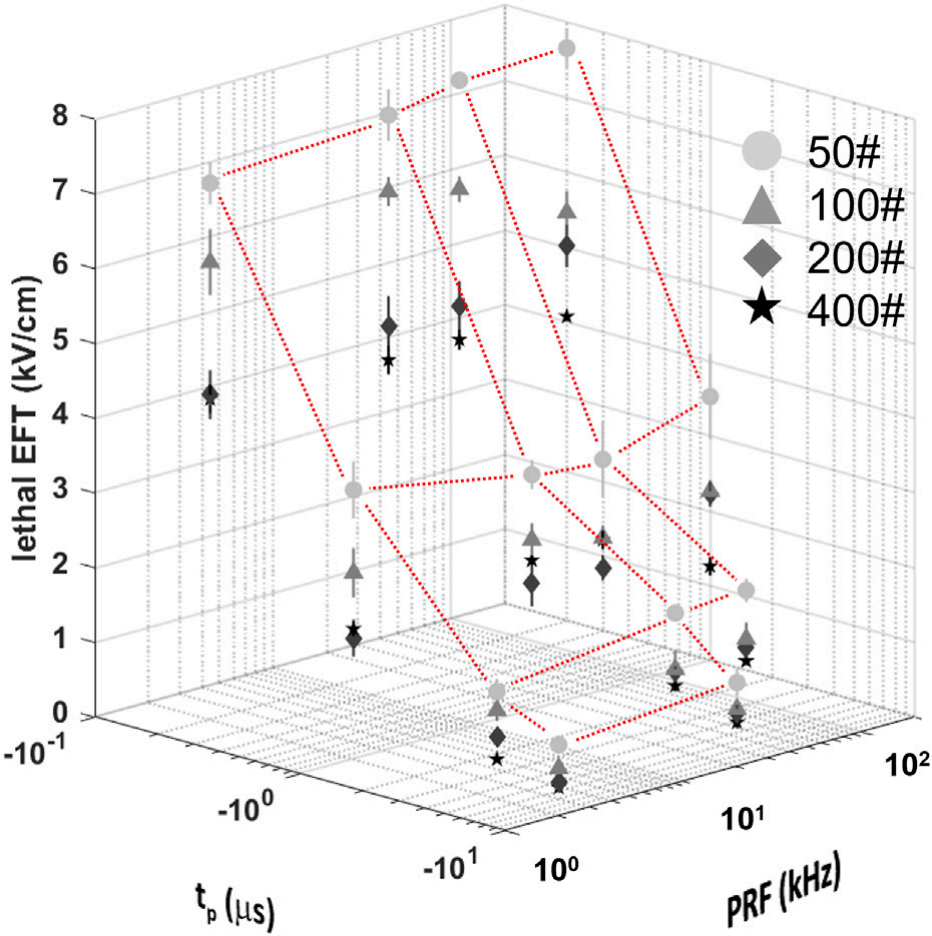
Lethal EFTs for a broad range of PEF waveform parameters in hiPSC-CMs. Lethal EFTs for all the combinations of PEF parameters tested. Trains of 50, 100, 200 and 400 pulses (circles, triangle, diamonds, stars, respectively) and t_p_ = 0.2, 1, 5, and 10 µs were delivered at PRF = 2, 20, 50, 200 kHz to hiPSC-CM monolayers. EFTs were calculated from cell death areas that were quantified from the borders of Calcein-AM staining 2–4 h after PEF treatments. Red dotted lines highlight the dependent variable space studied, 50# was used as example. See text for more details. The error bars represent the standard error (95% confidence interval) for a sample size of *n* = 4–8.

Consistent with prior studies (Casciola et al., 2023), the analysis revealed that the lethal EFT was inversely proportional to both t_p_ and p# (Figure 3; Supplementary Figure S4). The most pronounced effect was observed for phase duration, with a ∼2-fold reduction in lethal EFT when t_p_ increased from 0.2 to 1 µs, and a 1.5-1.7-fold reduction when t_p_ increased from 1 to 5 and 10 µs. An increase in the number of pulses per train from 50 to 100 and from 100 to 200 resulted in a ∼1.2-1.4-fold and ∼1.1-fold decrease in lethal EFT, respectively. However, further increases in the number of pulses in the train did not produce a significant effect on the EFT.

The effect of the PRF was the least pronounced and showed fluctuations within a limited range. A comparison with previous results for 50 and 100 pulse trains indicated a strong agreement with the current data, despite the use of different pulse generators (Supplementary Figure S5). In general, trends from these two studies suggested a minor increase in EFT with higher PRF.

For instance, when evaluating hiPSC-CMs, an increase in t_p_ from 0.2 to 1 µs (*p*# = 50, PRF = 200 kHz) led to a decrease in lethal EFT from 7.66 to 3.53 kV/cm. Increasing the pulse number in the train resulted in a reduced lethal EFT, with values as low as 3.75 kV/cm for 400 pulses per train (t_p_ = 0.2 µs, PRF = 200 kHz). However, the lethal EFT increased only from 7.09 to 7.66 kV/cm when the PRF increased from 2 to 2,000 kHz (t_p_ = 0.2 µs, *p*# = 50).

To provide a quantitative description of the intricate relationships between the independent waveform parameters and EFT, a log-log fitting was developed using a machine learning algorithm which was explained in Section 2.6. Results from this fitting are described in Section 3.4.

### 3.3 Effect of PEF waveform parameters on adiabatic temperature increase

To assess the energy deposition resulting from the combinations of PEF parameters under investigation, the AD and AH were calculated at the lethal EFT, following the method described in previous studies (Ibey et al., 2009; Casciola et al., 2023). Our analysis revealed that variations in PEF parameters produced significant changes in AH, which ranged from approximately 4°C–19°C (Table 1; Supplementary Figure S6), corresponding to an AD range of 18–76 J/g, resulting in an absolute temperature increase from the baseline 37°C–41°C and 56°C.

Across all PEF waveforms tested, pulse trains with lower pulse numbers induced cell death at lower doses and resulted in less temperature increase, with values ranging from 5.1°C to 9.7°C for 50 pulses compared to 6.2°C–18.8°C for 400 pulses. When the number of pulses was held constant, a reduction in phase duration (t_p_) led to a decrease in temperature increase due to the lower energy deposition associated with shorter pulse applications.

### 3.4 Lethal EFT fitting by machine learning and the calculator tool base on HiPSC-CMs response to PEFs

A log-log regression was applied to fit the experimental data which has three logarithmic features. The fitting in this model was carried out by minimizing the residual sum of squares between the experimentally observed and model predicted natural logarithm of the lethal EFT (Hackeling, 2017). A 10-fold cross validation was performed to ensure the quality of the fit (Fushiki, 2011) and the coefficient of determination (*R* ^2^) for the training and test sets were calculated. The fitted log-log equation and all the corresponding calculated fitting parameters were reported in Table 3.

**TABLE 3:** Log-log fitting equation.

$c_{0}$	2.4178579179273973
$c_{1}$	−0.3311596538518343
$c_{2}$	−0.5105374744505103
$c_{3}$	0.0200345703712249

This table provides all the information that is required to calculate the lethal EFT in kV/cm using the following fitted equation: $EFT=\exp\left({c_{0}+c}_{1}\ln p_{\#}+c_{2}\ln t_{p}+c_{3}\ln f\right)$ , where EFT is the lethal EFT in kV/cm, $p_{\#}$ is the number of pulses, $t_{p}$ is the duration of the pulse in µs, f is the PRF in kHz, and $c_{i}$ are fitting coefficients taken from this table.

**FIGURE 4:**
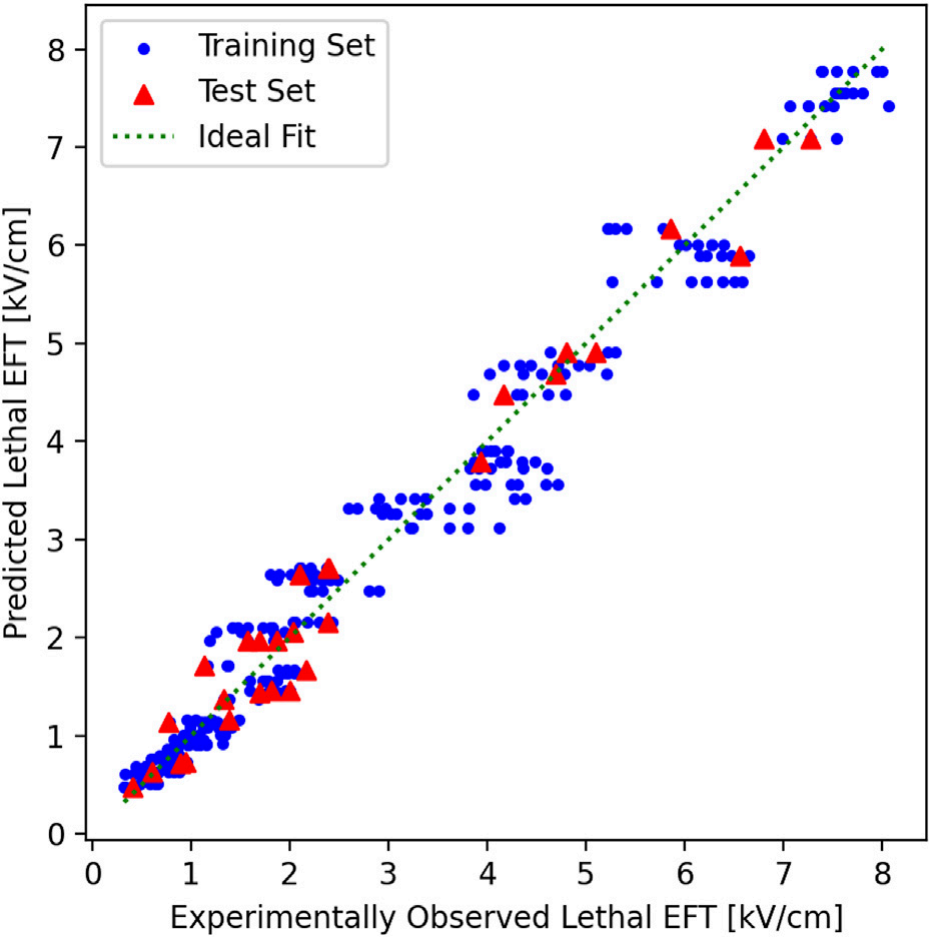
Predicted vs. experimentally observed EFT in training and test sets. In the 10-fold cross validation, the experimental results were randomly shuffled and split to 10 groups. In each cross-validation fold, one group was chosen and held as the test set (red triangles), and the rest of the groups as training (blue circles) and test sets (red triangles). The plot here shows the results from the last fold of the 10-fold cross-validation. The training set here was used to fit the model and provide the final log-log fitting. The log-log fitting was used to generate a prediction, based on the independent parameters for each point, of both the training and the test/validation tests. The predicted lethal EFT was plotted vs. the experimentally observed lethal EFT. In an ideal fitting (green dotted line), the predicted and experimentally observed values must be identical. Both the training and test sets are clustered closely around the ideal fitting line. Notice that the points are closer to the ideal fit line for smaller experimentally observed lethal EFTs and their distance remains less than 33% of the lethal EFT at larger experimentally observed lethal EFTs which keep the maximum relative error below 33% for all values.

The *R* ^2^ scores for the training sets were 0.954 ± 0.002 and the test sets 0.949 ± 0.017. The maximum absolute fitting error was 1.16 kV/cm at the predicted lethal EFT of 3.56 kV/cm which also led to the maximum relative error of 33%. The predicted and experimentally observed lethal EFTs in the training and test sets against the ideal fitting line show a good agreement (Figure 4). In an ideal fit, the predicted and experimentally observed values are identical. It is noticed that model predictions in the training and test sets are clustered closely around the ideal fit line which indicates the high quality of the fitting.

**FIGURE 5:**
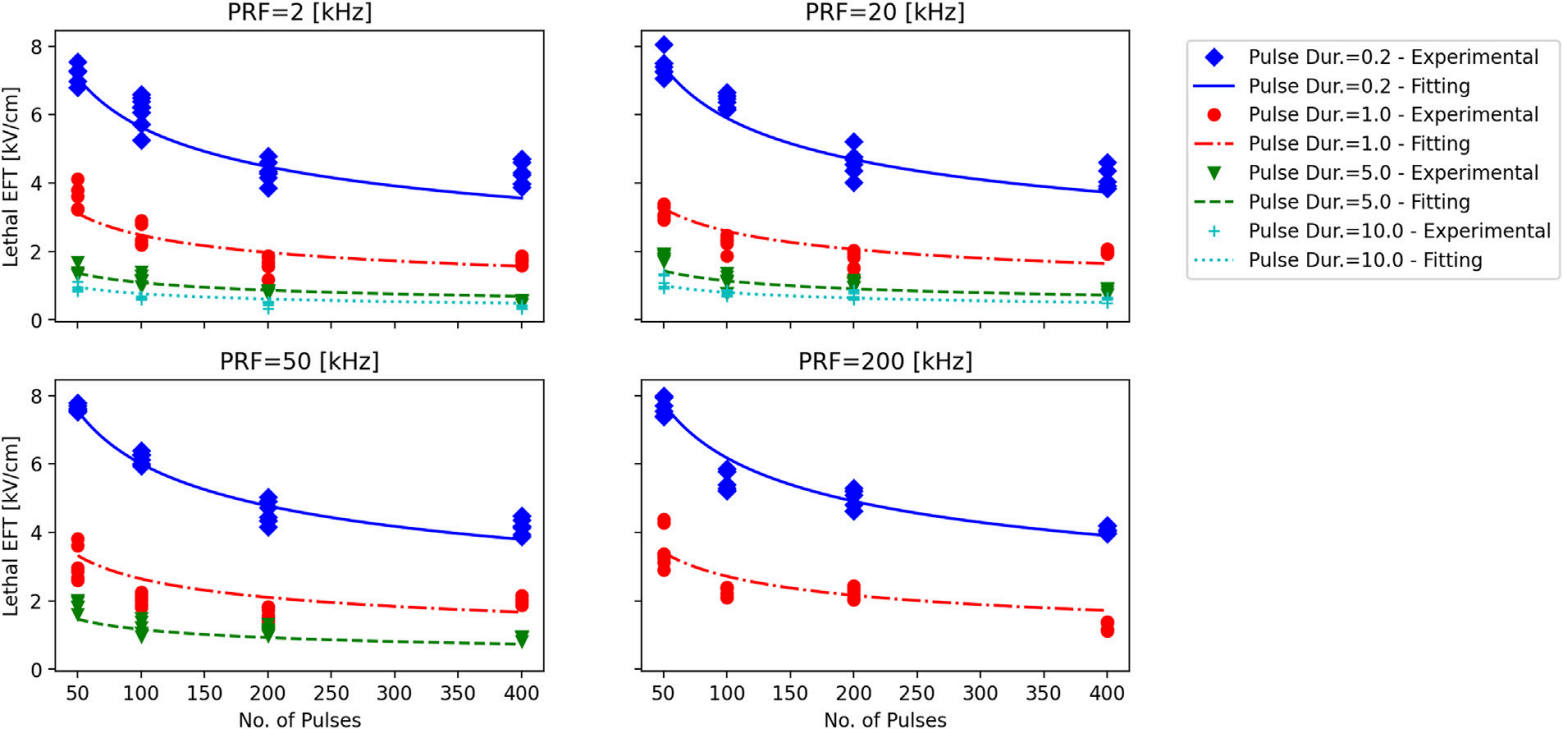
Experimentally observed lethal EFT vs. the results of log-log fitting. Each subplot corresponds to a PRF that is displayed above the subplot. Trains of 50, 100, 200 and 400 pulses with phase durations of t_p_ = 0.2, 1, 5, and 10 µs (diamonds, circles, inverted triangles, and plusses) were delivered to hiPSC-CM monolayers. Lethal EFTs were calculated from cell death areas that were quantified from the borders of Calcein-AM staining 2–4 h after PEF treatments. Experimentally observed EFTs for each case are compared against the log-log fitting results. Notice the smooth transition in the fitting lines.

The experimentally observed lethal EFTs against the fitted trend lines for various number of pulses, phase durations, and PRFs was reported in Figure 5. The fitted lines follow the experimental results smoothly, without any spurious minimums or maximums. The autocorrelation analysis reveals that as phase duration and the number of pulses increased (−0.69 and −0.29 correlation coefficients, respectively), the EFT decreased, while it exhibited an increase with higher PRF (0.27 correlation). Furthermore, this analysis provides supporting evidence that phase duration exerts the most pronounced effect on EFT, followed by the number of pulses and PRF, in respective order.

**FIGURE 6:**
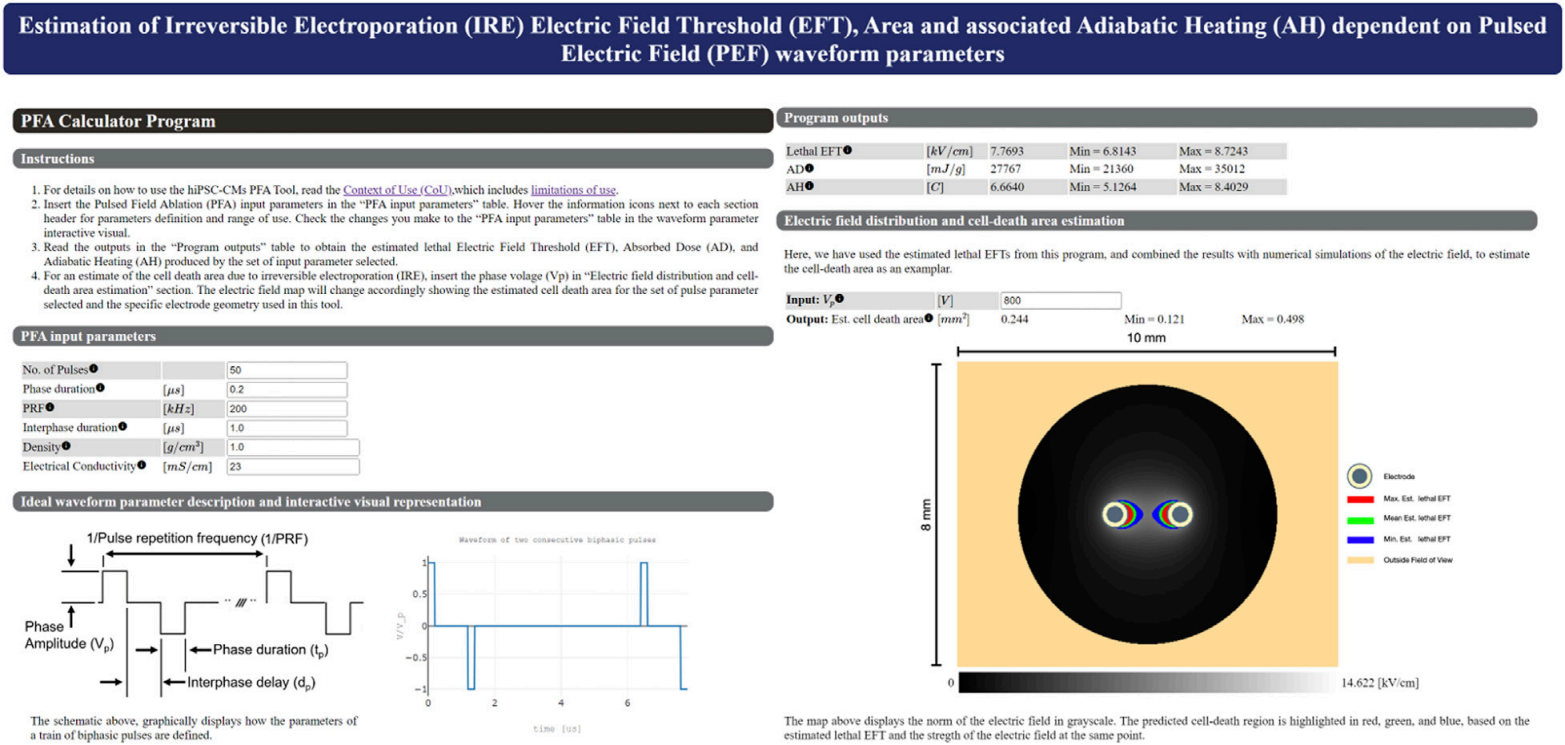
Main graphical user interface of the HTML-based tool that predicts the lethal EFT from the number of pulses, phase duration, and PRF. The user can easily enter the independent parameters in the graphical user interface and receive an estimate for the dependent parameters. Density and Electrical conductivity are specific to the user experimental set up and are used for Peak SAR, AD and AH calculations. If the entered parameters are out of the experimental range of this study, the program will display an warning message that indicates which parameter is out of range and revert back to the closest acceptable input. This ensures that users are only provided with predictions that are within the scope of the experiments. The program also provides the estimated lesion area using the numerical simulation results for our experimental electrode setup given an input voltage, i.e., based on the lethal EFTs.

When the performance of the log-log fit was established, it was subsequently used to create a web-based calculator tool that can estimate the EFT based on the input number of pulses, phase duration, and pulse repetition frequency (Figure 6). The calculator tool is a web-page that serves as the graphical user interface (GUI) that provides easy interactions with the program (GitHub - dbp-osel/PFACalculatorTool: This tool is developed to estimate the lethal PFA from input pulse parameters). The error estimates are provided for each given output. The input parameters are checked to ensure that they lie within the experimental range. If the inputs are out of the experimental range of this study, the program displays a warning message and changes that input to the closest acceptable value to provide the lethal EFT predictions. The warning message clearly indicates which parameter was out of range. This ensures that the outputs are interpolated values only and no extrapolation is carried out.

## 4 Discussion

The development of novel medical devices, particularly those in the field of electrophysiology, has undergone a remarkable transformation in recent years. Pulsed Field Ablation (PFA), an emerging technology, has garnered considerable attention as a promising technique for cardiac and tissue ablation. As PFA continues to evolve and attract interest from the medical community, the importance of robust nonclinical testing methods cannot be overstated. Nonclinical testing methods are indispensable for assessing the performance and safety of PFA devices at an early stage of their development. These methods offer a strategic approach to de-risking the path to clinical implementation. By subjecting PFA devices to rigorous evaluations in controlled environments, researchers and developers can identify potential issues, refine device designs, and ultimately enhance their overall effectiveness.

Determining the lethal Electric Field Thresholds (EFT) is a key challenge in optimizing PFA treatments, as this parameter varies with PEF waveform characteristics. In this study, we improved our previously developed laboratory protocol using hiPSC-CM in monolayer format to evaluate the impact of a wide range of clinically relevant biphasic pulse parameters on lethal EFT and adiabatic heating (AH) (Casciola et al., 2023). Three key advancements are implemented in our current study compared to Casciola et al. (2023). Firstly, the proposed assay for assessing iPSC-CM response to PEF waveform parameters was enhanced by refining the method for distinguishing between live and dead cells using different dyes. This refinement results in a more robust assessment of cell death, thereby enhancing the reliability of our findings. Secondly, we have expanded the scope of pulse parameters investigated in our current research to include clinically relevant values, providing a more comprehensive understanding of the impact of PEF waveform characteristics on cell viability. Lastly, a significant advancement is the development of a predictive calculator based on machine learning. This tool analyzes various parameter combinations to accurately forecast the electric field threshold for cell death, even across interpolated values. Our results shed light on the complex relationship between PEF parameters and lethal EFT in hiPSC-CMs and have implications for the advancement of PFA technology.

Our findings demonstrate a strong correlation between cell death in hiPSC-CMs and the number and duration of pulses in each train. Of note, the phase duration exerts a significant influence on lethal EFT, with longer phase durations associated with a decrease in EFT. Our study provides quantitative evidence to support the notion that extending the phase duration can enhance the efficacy of PFA treatments by lowering the EFT, potentially minimizing side effects while achieving effective ablation.

The number of pulses within each train also plays a crucial role, with an increased number of pulses resulting in a reduced lethal EFT. This finding implies that modifying the number of pulses in a PFA treatment can be a strategy for optimizing the procedure. However, it is worth noting that the effect of pulse number may saturate beyond a certain point, as further increases did not significantly affect the EFT while producing substantial thermal increase. Therefore, striking a balance between pulse number and phase duration is essential to achieving the desired ablation while avoiding excessive energy deposition.

In contrast to phase duration and number, pulse repetition frequency showed a comparatively weaker influence on lethal EFT. While it exhibited a positive correlation with EFT, comparable to García-Sánchez et al. (2022); Gudvangen et al. (2022), the effect was less pronounced. This suggests that adjusting PRF may have a lesser impact on the efficacy of PFA treatments, as compared to other pulse parameters. Additionally, in our study, AH does not account for heat dissipation and represents a worst-case scenario in temperature increase, which could potentially be mitigated by lower PRF. PEF treatment-induced heating should be minimized to avoid cell death by thermal damage, particularly when the temperature increase is sustained over time. High absorbed doses can lead to a non-negligible temperature rise, emphasizing the importance of avoiding excessive heating in the planning of PEF treatments.

Furthermore, the development of a calculator, based on machine learning algorithms and our experimental data, has the potential to revolutionize nonclinical safety and performance assessment. The primary objective of the hiPSC-CM PFA Tool is to assist investigators with quantifying irreversible electroporation (IRE) and thermal increase of PEF typical of PFA treatments *in vitro* using human cardiomyocyte models in monolayer format. The hiPSC-CM PFA Tool provides an online calculator that enables the user to promptly obtain information on an estimated EFT for cell death and adiabatic temperature increase expected for a combination of PFA waveform parameters. It should be noted that the estimation error of the PFA calculator tool remains bound and is not more than 30% for the predicted values. Additionally, it should be noted that the errors are of the same order of magnitude as the uncertainty and variability in the experimental results which can be up to ±35%. Therefore, we suggest that the estimated range be taken into account rather than the mean estimated value. If the waveform parameters intended to be tested is outside the range investigated in this tool, the user can rapidly generate the appropriate *in vitro* environment to perform PFA studies as described here and in Casciola et al. (2023). This tool serves as a non-clinical platform to provide safety and performance assessment of PEF applied to a human cardiac model, facilitating early-stage technology development, de-risking product development and decision making through a clinically relevant model. This tool is a simple and straightforward package that leverages standard laboratory equipment and technical capabilities relative to complex animal studies, and is intended to be used by medical device developers, academia, and contract research organizations (CROs). The user will have the capability to input various parameters targeted for testing and receive estimates to be able to evaluate which parameter combinations may minimize both EFT and AH. This curated set of pulse parameters can subsequently undergo validation on a limited number of animal models. Furthermore, when coupled with numerical modeling that incorporates the specific electrode geometry and anatomical target site, the user will be empowered to estimate alterations in ablation volume (Kos et al., 2023), corresponding to changes in pulse parameters, utilizing EFTs obtained from this tool.

While this study provides valuable insights into lethal EFT in hiPSC-CMs and its dependence on various PEF parameters, it has some limitations including the ones already described in Casciola et al. (2023). Firstly, we restricted our study to biphasic pulses, although short uniphasic pulses delivered in bursts have shown to offer advantages in terms of the reduction of neuromuscular effects and mitigation of thermal effects (Gudvangen et al., 2022). Secondly, this method cannot take into account the possibility of neuromuscular stimulation, which could potentially influence the choice of specific parameters, such as the PRF (Gudvangen et al., 2022). This is one of several ways in which the use of hiPSC-CMs, while minimizing species and biological disparities, does not fully replicate the complex *in vivo* environment of the heart. Further, our assay is developed based on cell monolayers that lack the complexity of 3D models, thus studies in animal models are necessary to validate the efficacy of the developed open-source calculator in real-world applications. In this study, we utilized rod electrodes, which are a simplified version of more intricate realistic catheter geometries. While it is expected that the lethal EFT remains unchanged, we aim to validate this hypothesis and explore the impact of electrode geometries on the volume of cell death in future works. Finally, the log-log fitting is an attempt to best fit the experimental results by minimizing the residual sum of the squares. It should be noted that this log-log fitting was not designed from physics’ first principles, and a model that explains the intricate dependencies of the lethal EFT based on first principles is yet to be proposed. Furthermore, the log-log fitting is carried out to be used only for interpolation. The fit was not tested for extrapolation and extension of the range of parameters of the study. The uncertainties in the log-log fitting outputs stem from the inherent uncertainties present in the experimental data.

## 5 Conclusion

This regulatory science research has introduced a standardized nonclinical assay that uses human induced pluripotent stem cell-derived cardiomyocytes (hiPSC-CMs) to evaluate lethal electric field thresholds (EFT) and adiabatic heating (AH). The results demonstrate the ability of pulsed electric fields (PEFs) to induce cell death in hiPSC-CMs and establish a clear link between pulse parameters and cell response.

A significant outcome of this research is the development of an open-source online calculator, which, once validated, holds great promise in early-stage Pulsed Field Ablation (PFA) device de-risking and performance assessment. This tool allows developers to efficiently compare estimated outcomes of different pulse parameter combinations, aiding in the selection of a limited set of waveforms for testing. Additionally, when used in conjunction with numerical modeling of specific catheter geometries, the tool can generate predictions of variations in ablation volumes and temperature increases associated with the chosen waveforms. Importantly, this predictive capability reduces the reliance on animal models, streamlining development and enhancing efficiency.

The impact of this tool extends beyond the laboratory, as it has the potential to inform regulatory decision-making regarding cardiac ablation medical devices. By providing standardized, data-driven assessments, regulators can evaluate the safety and efficacy of PFA devices. This has the potential to accelerate the transition from innovation to clinical implementation, ultimately benefiting patient care. In fact, while our study primarily targets optimizing PFA during the early stages of device development, its clinical significance lies in ensuring safer and more effective treatments and outcomes from the resulting optimized devices.

Future research efforts will focus on validating these findings in actual tissues.

## Data Availability

The original contributions presented in the study are included in the article/Supplementary Material, further inquiries can be directed to the corresponding authors.
