# Supplementary material for: Pulsed electric field performance calculator tool based on an in vitro human cardiac model
Source: Front Physiol. 2024 Jun 7;15:1395923. doi: 10.3389/fphys.2024.1395923 (PMC11190366; doi:10.3389/fphys.2024.1395923)
Supplement: Supplementary file 1 [file DataSheet1.DOCX]

Supplementary Material

# Supplementary Figures

## Human induced pluripotent stem cell-derived cardiomyocytes (HiPSC-CMs) staining, electroporation, and imaging timeline.


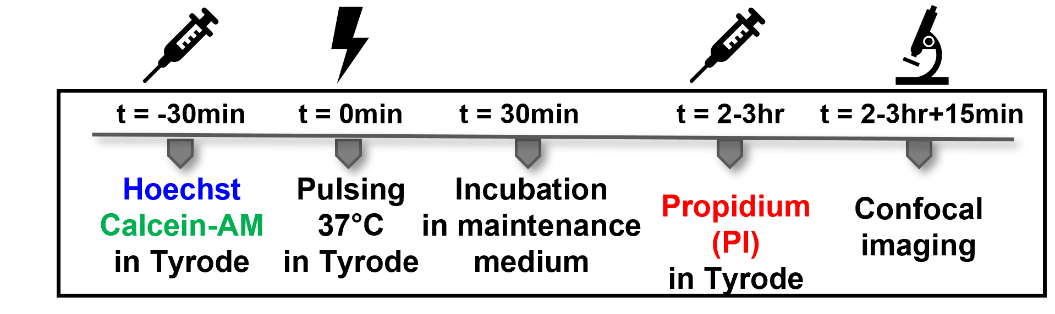


**Supplementary Figure 1.** Hoechst and Calcein-AM were added to the modified Tyrode solution 30 min prior to PFE treatments. 30 min after treatment, cells were moved to a 37°C 5% CO_2_ incubator in iCM-SF. During this time, cells reversibly electroporated repaired. Fifteen minutes prior the 2-3 h imaging, iCM-SF was substituted by modified Tyrode solution containing PI staining permanently damaged cell membranes.

## Comparison of Electric Field Threshold (EFT) values in human induced pluripotent stem cell-derived cardiomyocytes (hiPSC-CMs) staining calculated from Calcein-AM and Propidium Iodide staining


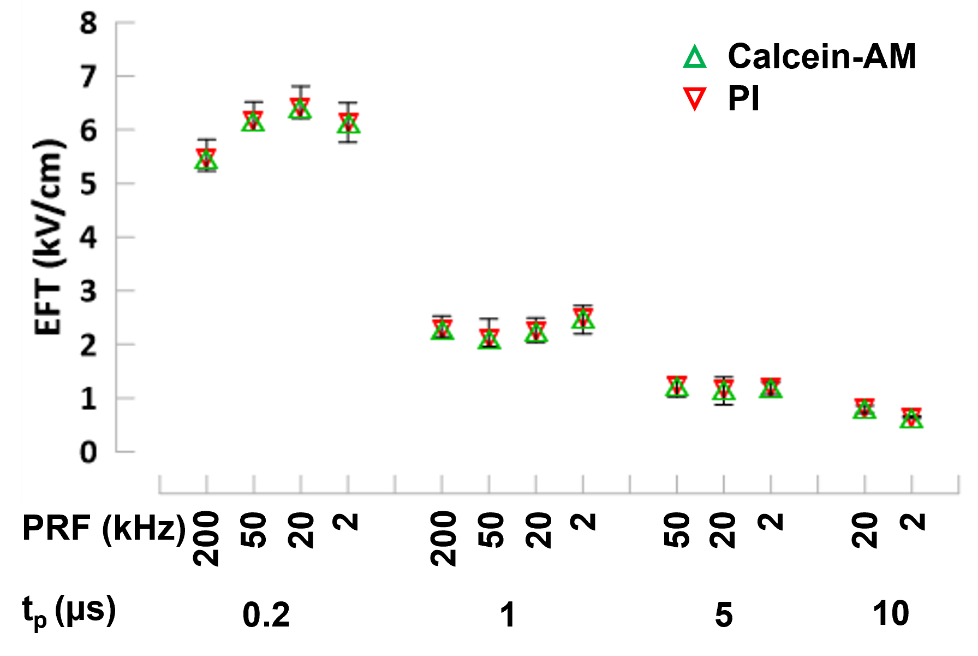


**Supplementary Figure 2.** For the same data set, cell death areas were assessed from Calcein-AM, and Propidium Iodide (PI) staining, green and red triangles, respectively. The Pulsed Electric Field (PEF) trains applied for different pulse repetition frequencies (PRF) and phase durations (t_p_) contained 100 bipolar pulses. n = 2-7.

## Representative Calcein-AM and Propidium Iodide staining assessing cell death in human induced pluripotent stem cell-derived cardiomyocytes (hiPSC-CMs) for different Pulsed Electric Field (PEF) waveforms


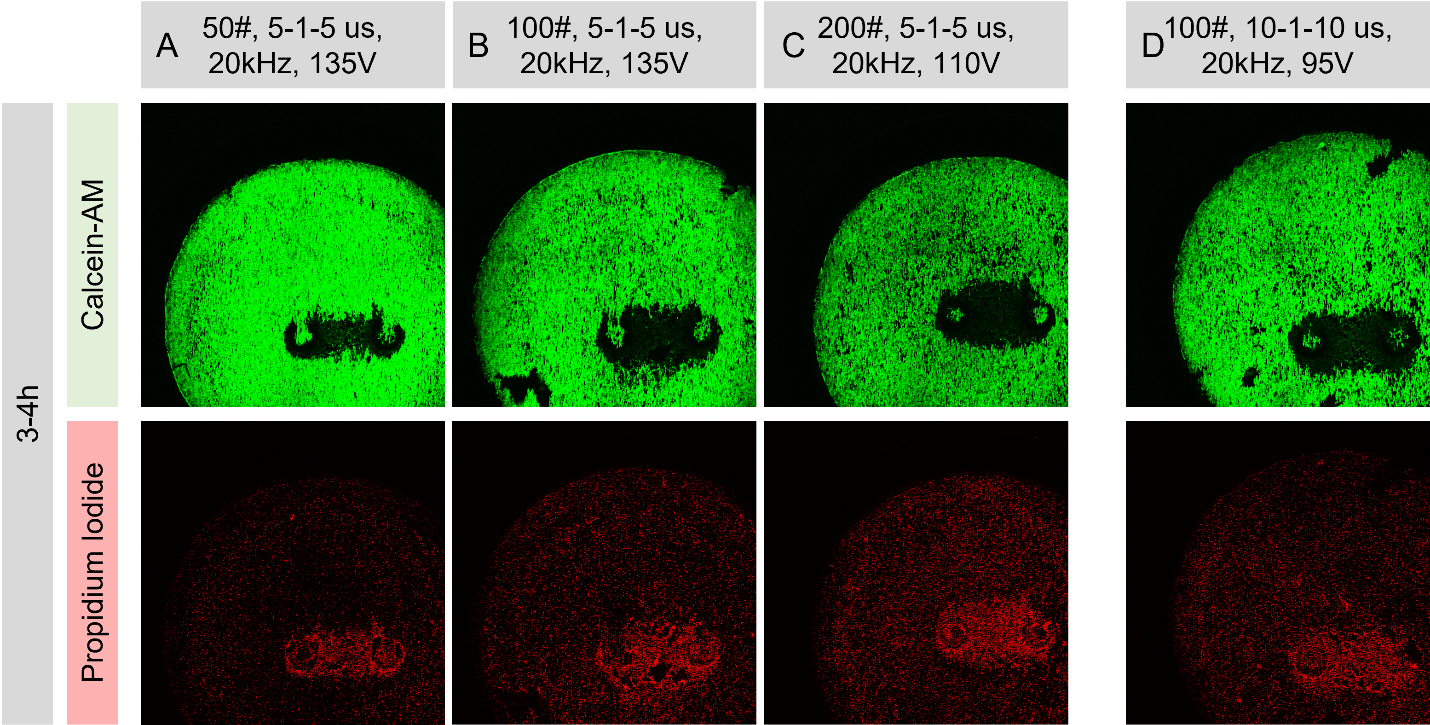


**Supplementary Figure 3.** Representative fluorescent images, Calcein-AM and Propidium Iodide top and bottom panels, respectively, are shown for phase duration t_p_ 5 and 10 µs, pulse repetition frequency PRF 20 kHz, pulse number p# 50, 100, 200. The phase amplitude V_p_ was varied to target a detectable effect while avoiding detachment in hiPSC-CMs.

## Lethal Electric Field Thresholds (EFTs) for a broad range of Pulsed Electric Field (PEF) waveform parameters in human induced pluripotent stem cell-derived cardiomyocytes (hiPSC-CMs)


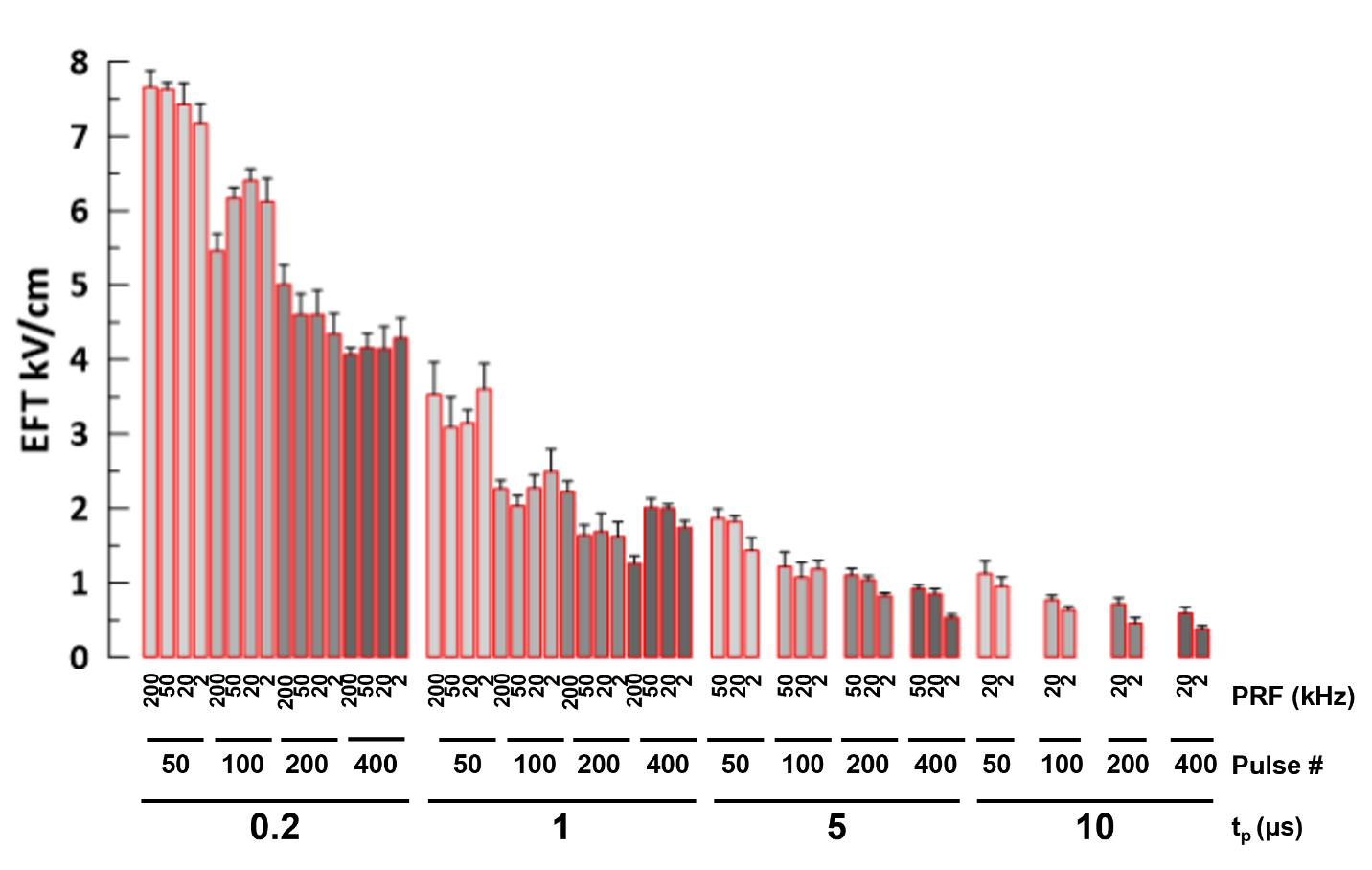


**Supplementary Figure 4.** Lethal EFTs for all the combinations of PEF parameters tested. Trains of 50, 100, 200 and 400 pulses and phase duration t_p_ = 0.2, 1, 5, and 10 µs were delivered at pulse repletion frequencies PRF = 2, 20, 50, 200 kHz to hiPSC-CM monolayers. EFTs were calculated from cell death areas that were quantified from the borders of Calcein-AM staining 2-4 hours after PEF treatments. The error bars represent the standard error (95% confidence interval) for a sample size of n = 4–8.

## Lethal Electric Field Thresholds (EFTs) in human induced pluripotent stem cell-derived cardiomyocytes (hiPSC-CMs)

## comparing results from the current study and [1]


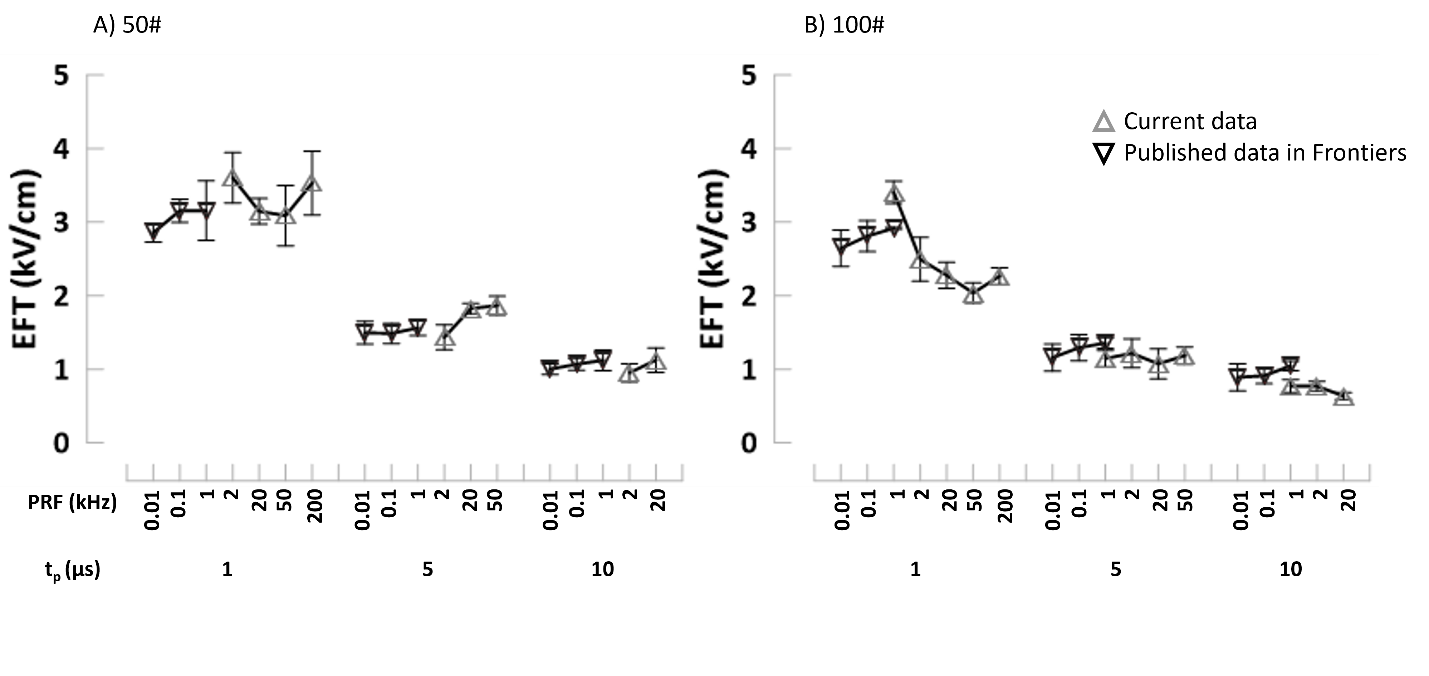


**Supplementary Figure 5.** Lethal EFTs for a subset of Pulsed Electric Field (PEF) parameters tested. Trains of 50, and 100 pulses and t_p_ = 1, 5, and 10 µs were delivered at pulse repetition frequencies PRF = 0.01, 0.1, 1, 2, 20, 50, 200 kHz to hiPSC-CM monolayers. EFTs were calculated from cell death areas that were quantified from the borders of Calcein-AM staining for the current data and of Propidium Iodide from [1]. The error bars represent the standard error (95% confidence interval).

## Calculated Adiabatic Heating at the Electric Field Thresholds (EFTs) for cell death in human induced pluripotent stem cell-derived cardiomyocytes (hiPSC-CMs)


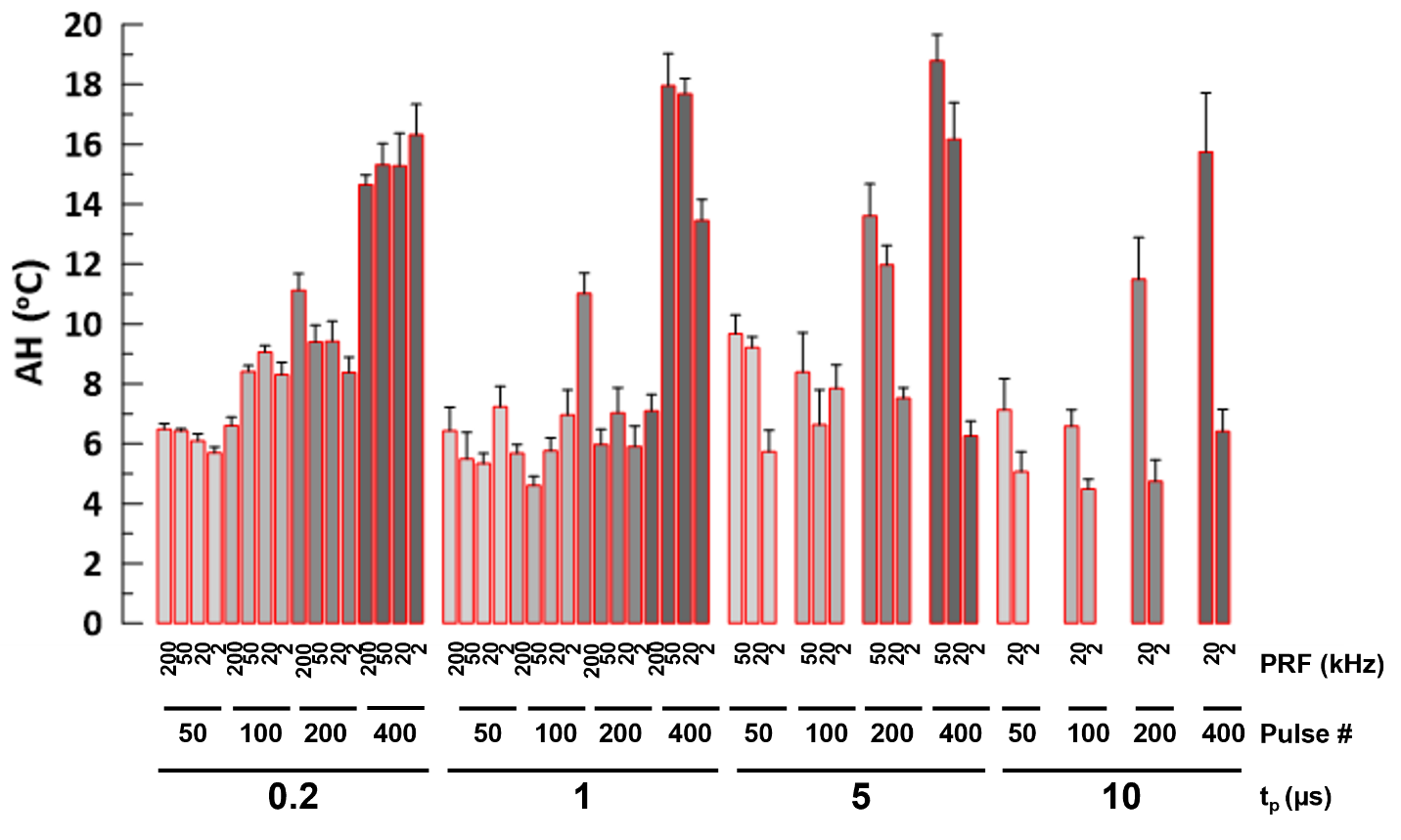


**Supplementary Figure 6.** Adiabatic heating at the EFT for cell death in hiPSC-CMs calculated for all the combinations of Pulsed Electric Field (PEF) parameters tested. Trains of 50, 100, 200 and 400 pulses and phase duration t_p_ = 0.2, 1, 5, and 10 µs were delivered at pulse repetition frequencies PRF = 2, 20, 50, 200 kHz to hiPSC-CM monolayers. The error bars represent the standard error (95% confidence interval) for a sample size of n = 4–8.

# References

[1] M. Casciola, T. K. Feaster, M. J. Caiola, D. Keck, and K. Blinova, "Human in vitro assay for irreversible electroporation cardiac ablation," *Frontiers in physiology*, vol. 13*,* p. 1064168doi: 10.3389/fphys.2022.1064168.
